# Hippocampal and Cerebellar Involvement in Opioid-Associated Amnestic Syndrome: A Unique Case Report

**DOI:** 10.7759/cureus.44248

**Published:** 2023-08-28

**Authors:** Evan McConnell, James Bai

**Affiliations:** 1 Imaging Science, University of Rochester Medical Center, Rochester, USA

**Keywords:** hippocampal restricted diffusion, cerebellar restricted diffusion, opioid-associated amnestic syndrome, anterograde amnesia, oas, fentanyl-associated toxic encephalopathy, opioid-induced amnesia

## Abstract

We present a case of opioid-associated amnestic syndrome (OAS) in a 46-year-old female with persistent acute anterograde amnesia and biochemically confirmed with fentanyl use. The brain Magnetic Resonance Imaging (MRI) examination demonstrated symmetric restricted diffusion and T2/fluid-attenuated inversion recovery (FLAIR) hyperintensities in the hippocampi and cerebellum. While cases of cerebellar findings in OAS are rare in the literature, this is a unique case with corresponding images that demonstrate cerebellar involvement in addition to the more common hippocampal finding.

## Introduction

Opioid-associated amnestic syndrome (OAS) is a rare condition that has only been recently recognized, with only 40 cases documented in existing literature [1,2]. It is clinically manifested by acute onset of antegrade memory loss in the setting of opioid use, most frequently associated with fentanyl. The characteristic imaging features consist of bilateral hippocampal edema shown as symmetric hypoattenuation on Computed Tomography (CT) and bilateral hippocampal restricted diffusion on diffusion-weighted imaging (DWI) with associated hyperintense signal abnormalities on fluid-attenuated inversion recovery (FLAIR) and T2-weighted sequences on MRI. Barash et al. have proposed specific diagnostic criteria for OAS, which require the presence of new-onset amnesia greater than 24 hours in duration, a positive toxicology test for opioids, and evidence of bilateral hippocampal injury on CT or MRI [2].

Although the exact pathophysiological mechanism of OAS remains uncertain, two possible mechanisms have been suggested. One hypothesis involves cerebral ischemia caused by hypoxia or opioid-induced reversible vasospasm [3]. The alternative hypothesis points to hippocampal hyperexcitability and hypermetabolism induced by opioid overdose, supported by evidence of fentanyl-induced neuronal hypermetabolism observed in the hippocampus of rats [4,5]. 

## Case presentation

A 46-year-old female with a past medical history of polysubstance abuse presented to the emergency department with altered mental status, having been found unconscious. After administration of intranasal naloxone, her mental status improved. The toxicology screen test was positive for fentanyl, confirmed with liquid chromatography-tandem mass spectrometry. Other laboratory tests, including complete blood count (CBC), basic metabolic panel (BMP), hypercoagulability workup, and thyroid stimulating hormone (TSH) were all within normal limits. Testing for herpes simplex virus (HSV), human immunodeficiency virus (HIV), and syphilis all returned negative results. The patient had persistent encephalopathy, experiencing anterograde amnesia, mild deficits in orientation, and moderate deficits in constructional ability, calculations, and reasoning. There were no other observable sensory or motor deficits in the patient.

**Figure 1:**
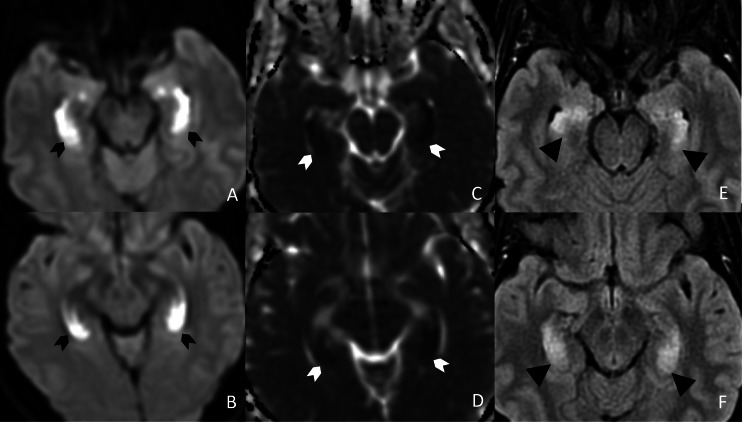
Opioid-associated amnestic syndrome (OAS) - cytotoxic edema in the hippocampi Axial DWI (A and B) and ADC map (C and D) demonstrate symmetric restricted diffusion of the bilateral hippocampal head, body, and tail with associated FLAIR hyperintensities on the axial FLAIR sequence (E and F). DWI:  Diffusion-weighted imaging; ADC: Apparent diffusion coefficient; FLAIR: Fluid-attenuated inversion recovery

**Figure 2:**
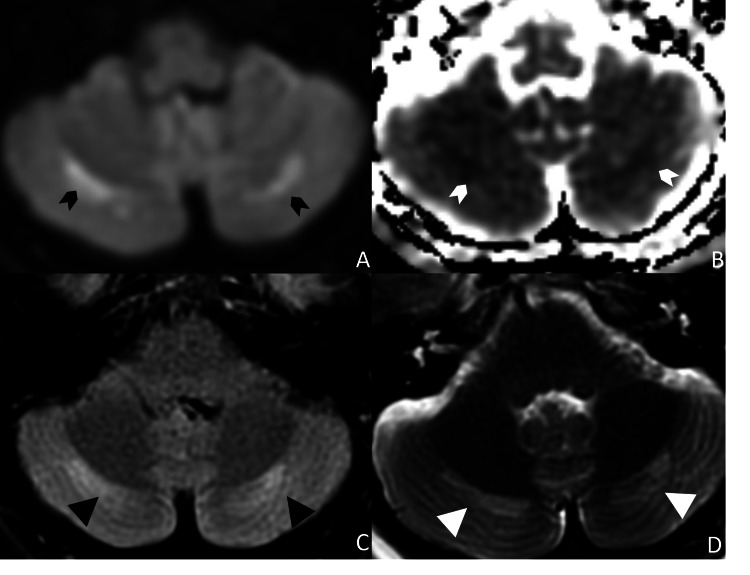
Opioid-associated amnestic syndrome (OAS) - cytotoxic edema in the cerebelli Axial DWI (A) and ADC map (B) demonstrated symmetric mild restricted diffusion of bilateral cerebelli with associated FLAIR and T2 hyperintensities on the axial FLAIR (C) and T2 (D) sequences. DWI: Diffusion-weighted imaging; FLAIR: Fluid-attenuated inversion recovery

Given these symptoms, further investigation was warranted, and a brain MRI without intravenous contrast was performed. The MRI exam demonstrated symmetric T2/FLAIR hyperintensities with abnormal restricted diffusion along the bilateral hippocampi (Figure 1). In addition to the hippocampal abnormalities, the exam also showed bilateral symmetric cerebellar T2/FLAIR hyperintensities with mild restricted diffusion (Figure 2).

The clinical presentation, toxicology test result, and imaging findings were consistent with OAS. Differential diagnoses include cerebellar, hippocampal, and basal nuclei transient edema, known as CHANTER syndrome, as well as hypoxic-ischemic encephalopathy. Transient global amnesia (TGA) can exhibit anterograde amnesia and hippocampal restricted diffusion (most commonly CA 1 region). However, TGA symptoms usually resolve within 24 hours, and the presence of symmetric hippocampal and cerebellar involvement makes this diagnosis unlikely in this case. Neither imaging nor clinical findings support heroin-induced leukoencephalopathy and posterior reversible encephalopathy syndrome (PRES). There are no other clinical or lab results that indicate alternative etiologies such as seizure, or central nervous system (CNS) infections including herpes encephalitis, or hypoglycemia.

Apart from the initial administration of intranasal naloxone, the patient also received intravenous thiamine, oral multivitamin, and acetaminophen. The patient received occupational and speech therapy as part of her supportive care. Over the course of her four-day hospital stay, the patient showed some improvement in anterograde memory formation and was subsequently discharged to home under the 24-hour care of a family member. Plans were made for brain rehabilitation and neuropsychiatric assessments in an outpatient setting. However, follow-up after discharge did not occur.

## Discussion

We present a case of OAS in a 46-year-old female with imaging findings of bilateral cerebellar involvement in addition to the classic hippocampal findings described in the diagnostic criteria proposed by Barash et al. in 2020 [4]. The hippocampi are highly metabolically demanding regions and are susceptible to damage caused by ischemia and toxic metabolic effect [6]. However, the exact mechanism of hippocampal injury in OAS remains uncertain [7]. It has been suggested that cerebral ischemia plays a significant role in the pathophysiology of OAS, although the direct neurotoxic effects of fentanyl may also contribute and could have overlapping effects [6]. The ultimate pathological manifestation is reduced diffusion in the extracellular space secondary to cytotoxic edema, shown as hyperintense signal abnormalities on diffusion-weight imaging and T2/FLAIR sequences.

The specific impact of fentanyl on the cerebellum's pathophysiology remains unclear. There has been a recently recognized condition known as CHANTER syndrome, which is linked to opioid toxicity. The syndrome is characterized by cytotoxic edema affecting the hippocampi, cerebellum, and basal ganglia, potentially leading to obstructive hydrocephalus due to cerebellar edema. To date, there hasn’t been a specific set of clinical and imaging criteria for this disease entity. It could potentially represent a more severe spectrum of OAS with a similar underlying mechanism [8]. Opioid-associated cerebellar edema has also been reported in the pediatric population after general anesthesia and use of fentanyl patch, known as pediatric opioid use-associated neurotoxicity with cerebellar edema (POUNCE) syndrome [9,10]. Also notably, cerebellar involvement is also seen in heroin-induced leukoencephalopathy, another opiate overdose-related encephalopathy, though heroin is a different class of opiate, and the route administration in this disease entity is through inhalation. The imaging pattern in heroin-induced leukoencephalopathy predominantly affects the cerebral and cerebellar white matter.

While this may be a unique case of coincidental cerebellar findings in a patient with OAS, it is plausible that because the cerebellum is not usually associated with amnestic states, the cerebellar findings in OAS have underemphasized leading to underreporting of cerebellar findings in OAS. This unique case of OAS, in addition to cerebellar findings observed in other opiate-related encephalopathies, raises the possibility of a link between acute opiate overdose and cerebellar involvement. Given the rarity and recent recognition of OAS, the long-term consequences remain somewhat uncertain. There are reports suggesting that amnestic symptoms resolved completely in some patients several months after an OAS diagnosis, but in other cases, severe symptoms endured. [11,12]. 

## Conclusions

This case highlights the cerebellar involvement, evidenced by symmetric bilateral T2/FLAIR white matter hyperintensities with restricted diffusion on imaging, in addition to the classic hippocampal findings in patients with OAS. Further studies and additional case reports of OAS could offer valuable insights into the underlying pathophysiology of fentanyl-associated toxic encephalopathy, the potential link between acute opiate overdose and cerebellar involvement, and aid in determining prognostic and therapeutic implications. 
